# *In Vivo* Bioluminescence Tomography for Monitoring Breast Tumor Growth and Metastatic Spreading: Comparative Study and Mathematical Modeling

**DOI:** 10.1038/srep36173

**Published:** 2016-11-04

**Authors:** Séverine Mollard, Raphaelle Fanciullino, Sarah Giacometti, Cindy Serdjebi, Sebastien Benzekry, Joseph Ciccolini

**Affiliations:** 1 Pharmacokinectics Laboratory, SMARTc Unit, Inserm S_911 CrO2, Aix-Marseille Univ, Marseille, France; 2 Pharmacology & Drug Development Group, Cancer Research UK Cambridge Research Institute, and Department of Oncology, University of Cambridge, Cambridge, United Kingdom; 3 MONC Team INRIA, Bordeaux France

## Abstract

This study aimed at evaluating the reliability and precision of Diffuse Luminescent Imaging Tomography (DLIT) for monitoring primary tumor and metastatic spreading in breast cancer mice, and to develop a biomathematical model to describe the collected data. Using orthotopic mammary fat pad model of breast cancer (MDAMB231-Luc) in mice, we monitored tumor and metastatic spreading by three-dimensional (3D) bioluminescence and cross-validated it with standard bioluminescence imaging, caliper measurement and necropsy examination. DLIT imaging proved to be reproducible and reliable throughout time. It was possible to discriminate secondary lesions from the main breast cancer, without removing the primary tumor. Preferential metastatic sites were lungs, peritoneum and lymph nodes. Necropsy examinations confirmed DLIT measurements. Marked differences in growth profiles were observed, with an overestimation of the exponential phase when using a caliper as compared with bioluminescence. Our mathematical model taking into account the balance between living and necrotic cells proved to be able to reproduce the experimental data obtained with a caliper or DLIT imaging, because it could discriminate proliferative living cells from a more composite mass consisting of tumor cells, necrotic cell, or inflammatory tissues. DLIT imaging combined with mathematical modeling could be a powerful and informative tool in experimental oncology.

Breast cancer is the most frequent spontaneous malignancy diagnosed in women and metastatic disease still bears a high mortality rate despite continuous efforts to improve clinical outcome^1^,^2^. It is estimated that there will be more than 400 000 new cases diagnosed every years in Europe, claiming approximately 90 000 deaths^3^. Despite current medical and technological advances, metastasis development remains a critical issue since advanced disease is associated with poor prognosis with most solid tumors. Metastasis has always been portrayed as the ultimate step of the progressing breast cancers; in fact 90% of mortality from cancer is attributable to metastases^4^,^5^. Recent evidence indicates that about a third of women diagnosed with small asymptomatic breast tumors (i.e., ~4 mm) already bear disseminated breast cancer cells in different organs^6^,^7^. Moreover, these occult micro-metastases can remain dormant for years before reemerging as potentially incurable secondary tumors as they can be surprisingly insensitive to adjuvant therapies that were originally effective against the primary tumor^4^,^6^. In this context, the study of metastatic spreading and the development of new strategies to address this issue remains an important challenge in experimental and translational oncology. The use of orthotopic murine models has been a very useful tool for studying breast cancer in translational research^8^,^9^. For decades, regular killing of the tumor-bearing animals to monitor the secondary lesions throughout time was the only way to evaluate and to understand such metastasis spreading. This strategy required an extremely high number of animals, thus infringing the “3 Rs rule” when conducting animal research. To address this issue, different non-invasive imaging technologies have been developed for years to visualize metastasis in living subjects. Developing a validated model to monitor in real-time metastatic spreading is critical to understand -and subsequently to identify relevant therapeutic strategies likely to prevent invasiveness or to eradicate already existing metastasis^10^. These include positron emission tomography (PET or microPET), single-photon emission computed tomography (SPECT or microSPECT), magnetic resonance imaging (MRI), ultrasonography (US), X-ray computed tomography (CT), and optical imaging (bioluminescence and/or fluorescence imaging). All of these techniques have pros and cons, and no single modality provides the ultimate tool to analyze the tumor evolution and the metastatic spreading during experimental therapeutics studies^11^. However, for preclinical studies, optical imaging is an excellent, time- and cost-effective method because of its simplicity, the short acquisition time, along with high resolution and elevated sensitivity required to address the issue of detecting small metastatic lesions throughout time. Moreover, this strategy is less potentially hazardous for the experimenters, because it does not require the handling of radioactive contrast agents and skips the issue of toxic-waste management. In preclinical cancer studies, bioluminescence and fluorescence imaging are increasingly used^12^,^13^. Bioluminescence is produced by cells that have been stably transfected with luciferase, thus allowing photon emission in presence of luciferine as substrate. This reaction requires ATP, thus ensuring that only living cancer cells will display light eventually. This is a crucial point since surrounding inflammatory tissues, necrotic tumor or calcified tissues can be mistakenly measured as tumor tissue upon most imaging techniques or standard volumetric measurement with a Vernier caliper. Emitted photons are scattered in the animal tissue throughout the whole body. Imaging systems recorded the spatial distribution of these scattered photons from the surface of the animal. Most of bioluminescent optical imaging systems provide 2-dimensional information in rodents^12^,^14^,^15^ with signal quantification by subsequent ROI (Region Of Interest) analyses on the animal surface. 2-D imaging is simple and provides a good but only relative measure of the internal light source brightness. Indeed, information’s on source depth and resulting attenuation through tissues are necessary to calculate the absolute source brightness measurement, which is not achievable with 2D approaches^16^. Recently, 3-dimensional (3D) capabilities have been made available, thus making possible to identify precisely the source localization, thus allowing subsequent tissue-attenuation corrections and absolute calculation of the actual source brightness. Diffuse Luminescent Imaging Tomography (DLIT) is a technique that analyzes images of the surface light emission from the animal to generate a 3-dimensional reconstruction of luminescent sources distribution inside the animal, including the smallest and deepest ones. This study aims at evaluating DLIT reconstruction reliability, as part of the monitoring of metastatic spreading in an orthotopic model of breast cancer, and to compare its performance with 2D bioluminescence imaging and canonical caliper-based measurement.

## Results

### Cells-to-light ratio determination

The amount of photons emitted by each tumor cell was evaluated as further described in the Methods section, by determining a calibration curve between a rising number of cells and the resulting increase in bioluminescent signal. Cell-to-light relationship was described by the following linear regression y = 88.4*x with r^2^ of 0.9988 (data not shown). Based on these data, an 88.4 photons/sec per cell relationship was established.

### DLIT reproducibility

**Figure 1:**
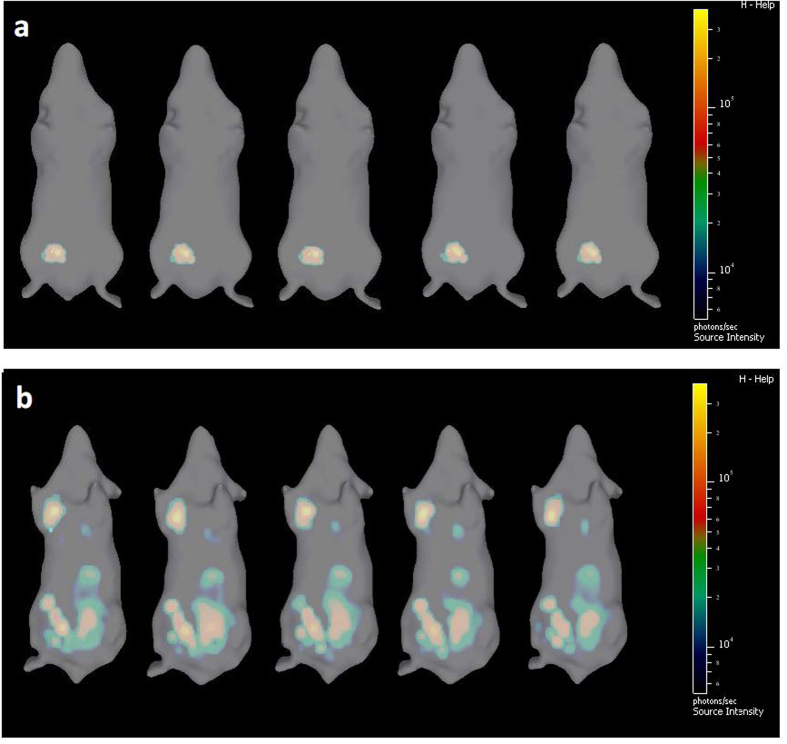
(**a**,**b**) Reproducibility of 3D-bioluminescence measurement. Bioluminescence tomography of mouse bearing (**a**) a small tumor or (**b**) an advanced stage tumor. For each condition, 10 reconstructions have been performed in a row. No statistical difference was evidenced between the reconstructions (p > 0.05, RM Anova).

For 3D reconstruction, the Living Image software undergoes different steps. This method uses *a priori* information about the optical properties of the subject to reconstruct the location and concentrations of internal bioluminescent sources from surface measurements. To each step of the reconstruction is associated a possible source of error called variability. To assess whether we could use the DLIT system for evaluation of tumor growth and metastatic spreading, 10 successive and independent reconstructions have been performed. For this purpose, were performed two reconstructions series of the same mouse in the beginning of the experiment (one week after the xenograft) and at end point with metastatic disease. Figure 1a displays the first series of acquisitions, 7 days after tumor engraftment. For the 10 reconstructions, the median value primary tumor was 2.9 × 10^8 ^photons/sec ± 1.6% (p > 0.05, Repeated Measure One-Way Anova). Figure 1b shows the same mouse bearing different metastatic sites at Day-43. Primary tumor exhibits a median value of 9.4 × 10^9^ photons/sec ± 3% of variation (p > 0.05, Repeated Measure One-Way Anova). Three main metastatic locations were observed: axillary lymph node (median value 4.4 × 10^9 ^photons/sec ± 3.6%), lungs (1.5 × 10^9^ ± 7.3%), and peritoneal (4.2 × 10^9^ ± 5.5%).

### DLIT reliability

After checking the DLIT reproducibility, the reliability and accuracy of the signal localization were tested. To assess the DLIT algorithm validity, the 3D signals were compared to those obtained by 2D ROI analyses and mice necropsy.

**Figure 2:**
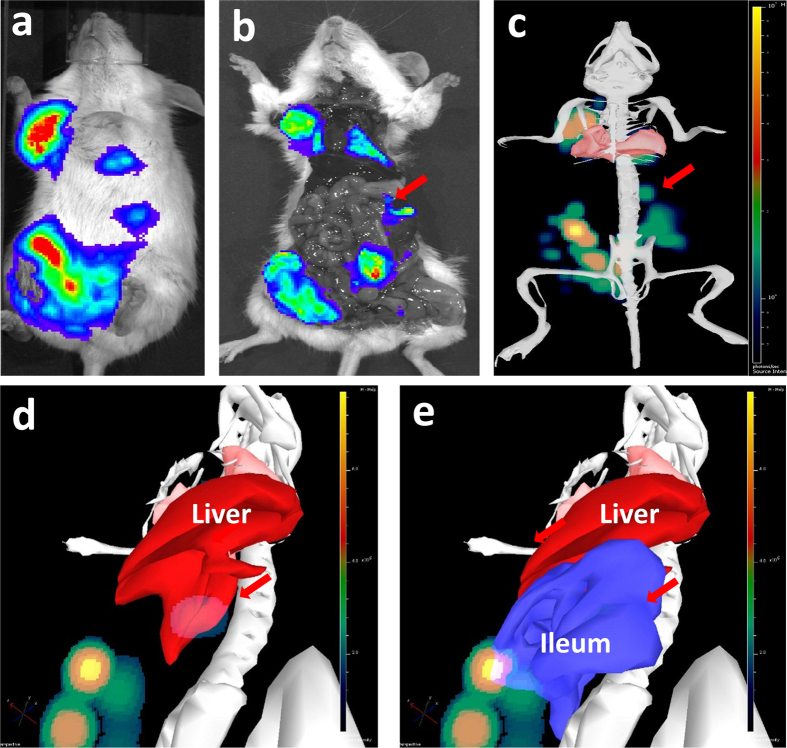
(**a**–**e**) Comparison between *in vivo* 2D imaging, 2D imaging after sacrifice and *in vivo* 3D imaging. Bioluminescence signals recorded from the same mouse 43 days after MDAMB231-Luc xenograft: *in vivo* 2D bioluminescence (**a**), 2D bioluminescence after mouse euthanasia (**b**) and *in vivo* 3D-bioluminescence imaging (**c**). Arrows: signals that could be wrongly attributed to liver metastasis were identified as peritoneal carcinosis upon DLIT reconstruction using the built-in organ atlas (**d,e**).

**Figure 3:**
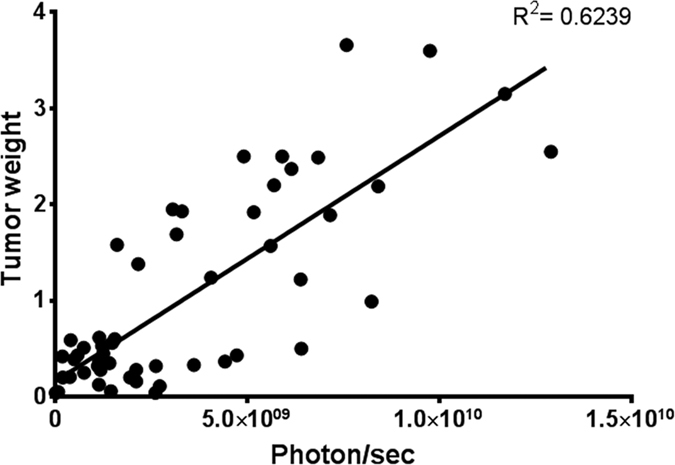
Correlation between tumor weight and 3D bioluminescence measurement. After necropsy, tumors were weighted and correlated with the last value recorded in photons/sec.

The 2D imaging at Day-43 post graft allows the distinction of 3 metastatic sites: the right axillary lymph node and one peritoneal plus one pulmonary metastatic sites, plus a possible liver metastasis (Fig. 2a). Another acquisition was done immediately after the mouse dissection, providing new information concerning the metastatic sites. This additional post-mortem acquisition showed that signals from primary tumor merge with close metastatic lesions in the lower peritoneal cavity, and that no liver metastasis was found. With standard bioluminescence imaging, discriminating primary breast cancer from nearby secondary lesion could only be achieved post-mortem by opening the mouse and placing it under the camera to avoid diffusion and merging of the 2D signals (Fig. 2b). Conversely, the 3D analysis and DLIT reconstruction using the built-in mouse anatomical atlas helped to discriminate and to localize all the sources before mice euthanasia (Fig. 2c). For instance, it was possible to establish that signal in the upper peritoneal cavity that could have been taken for a liver metastasis (red arrow) was actually a lesion on the ileum (Fig. 2d,e). With 3D imaging, the smaller metastasis (pulmonary) was detected with an intensity source less than 1 × 10^7 ^photons/sec (i.e. about 1 × 10^5^ cells). We also compared the number of photons measured by 3D imaging and the weight of tumor after its removal. A correlation (r^2^ = 0.62) between tumor weight and bioluminescence was found (Fig. 3).

### DLIT for metastasis spreading

**Figure 4:**
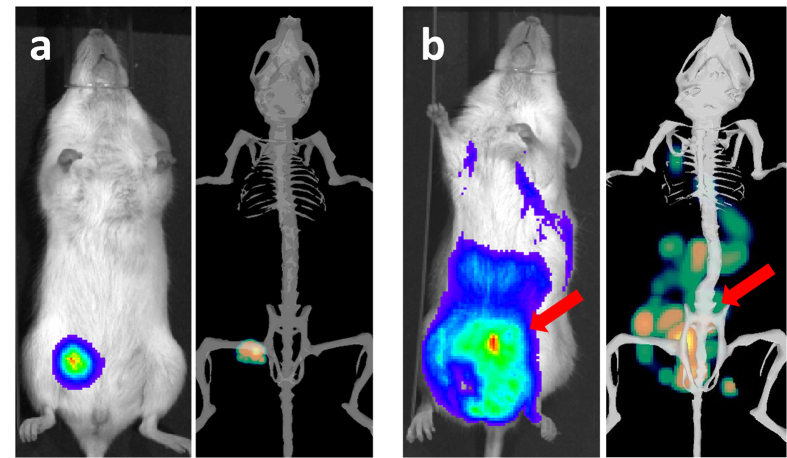
(**a**,**b**) Comparison between 2D and 3D imaging for localized (**a**) and advanced (**b**) tumors. Acquisitions were performed on the same mouse at day 9 (**a**), and day 43 (**b**). Red arrows represent the part of metastatic signal which is hidden by primary signal upon 2D imaging.

Only 2D bioluminescence and bioluminescent 3D tomography were compared to monitor metastatic spreading, because caliper can only address external measurements. At the beginning of the experiment, when primary tumors were small, 2D and 3D imaging showed similar results as displayed in Fig. 4a. Small tumors appeared as well defined and precisely localized regardless of the methods. Figure 4b shows a mouse with later extended metastatic spreading with multiple locations in the same area, i.e. primary tumor and peritoneal metastasis. The 2D analysis shows the primary tumor, and global metastasis at different sites: peritoneal, lungs, liver and lymph node, respectively, all merged in a global signal. The DLIT reconstruction allows a better distinction between the different metastatic sites: 1 axillary lymph node site, 1 lung site, 1 liver and 3 main peritoneal sites were detected in addition to the primary tumor. Of note, part of peritoneal metastasis signal was confounded with primary tumor in 2D, whereas 3D analysis allows a better resolution (Fig. 4b, red arrows).

### Primary Tumor Growth

**Figure 5:**
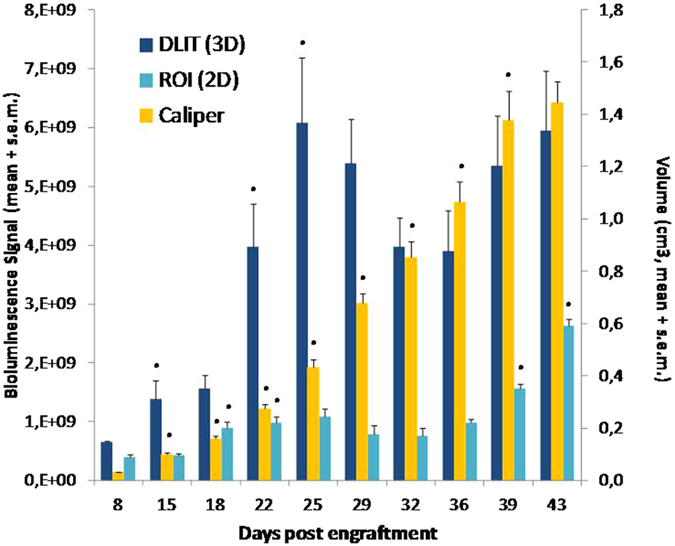
Tumor growth monitored using different procedures. The left axis represents the bioluminescence values recorded with 2D and 3D acquisitions and the right axis is the tumor volume measured by caliper and estimated with Carlsson formula^23^. Each bar is the mean + standard error recorded in 20 mice. *Statistically different from the previous measure (One-Way Anova with Tukey’s HSD Post Hoc multiple comparison testing).

Figure 5 shows evolution of the main tumor growth throughout time based upon monitoring using different methods: volumetric measurement of the external tumor using a Vernier caliper, 2D ROI-based measurement and 3D DLIT evaluation. Tumor external volume evaluated using caliper increased exponentially during the 35 first days post-graft, and finally seemed to reach a plateau of about 1,3 cm^3^ on Day-43. Growth curves were markedly different with either 2D or 3D bioluminescence imaging. Firstly, recorded signals were always higher with 3D imaging with mean signals 3.8-fold higher as compared with 2D acquisition for a same animal. Secondly, with both 2D and 3D bioluminescence monitoring, exponential phase only lasted 18–25 days before reaching a plateau. One-Way Anova with Tukey’s HSD Post Hoc multiple comparison testing showed that whether bioluminescence signals markedly increased during the early exponential phase (p < 0.05), no statistically significant increase was evidenced anymore from Day-25 to Day-43 (DLIT reconstruction) and from Day-18 to Day-36 (2D imaging). With 2D analysis, tumor growth resumed from Day-36 to Day-43 (p < 0.05, One Way Anova).

### Quantitative mathematical modeling analysis of tumor growth kinetics

**Figure 6:**
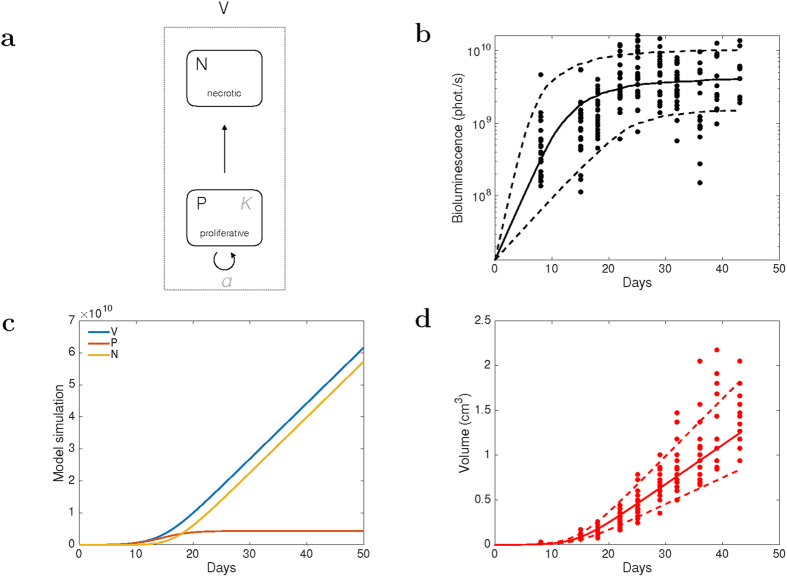
(**a**–**d**) Combined quantitative mathematical modeling of the bioluminescence emission and the caliper-measured volume kinetics. (**a**) Model scheme. (**b**) Model fit of the proliferative compartment to the 3D bioluminescence signal, using a population approach (mixed-effects) for inter-animal variability. Solid line = median. Dashed lines = 10^th^ and 90^th^ percentiles. Log scale. (**c**) Resulting model prediction of the total tumor volume, up to a proportionality constant λ and to appropriate conversion into cm^3^ (see text). (**d**) Simulation of the model dynamics with P = proliferative tissue, N = necrotic tissue, V = total volume. Parameter values are the typical parameter values as reported in Table 1.

To further inform on the volume and bioluminescence kinetics, we designed a mathematical model based on the hypothesis that the total volume is not only composed of proliferative tumor cells (as recorded by the bioluminescence), but also of necrotic tissue that would not be proliferatively active but nevertheless contribute to the caliper-measured volume while stopping to emit light signal. It assumes that the tumor volume (denoted by *V*(*t*) with *t* the time) is composed of two compartments (Fig. 6a). The first represents the living and proliferative cells, to be identified to the bioluminescence signal, and denoted *P*(*t*) Growth of the proliferative proliferation is assumed to be logistic, which expresses competition (for space or nutrients). Assuming a micro-environment carrying capacity *K* and a proliferation rate *a* (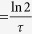, with *τ* the cell cycle length), the probability of cell division during a small time interval of length *dt* is 
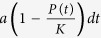
 and we have 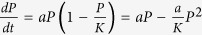. When tumor cells are unable to proliferate, they go to a necrotic compartment, denoted by *N*(*t*). Mathematically, these do not proliferate (and thus do not emit light) but still contribute to the total tumor volume. Writing a law of mass conservation expressing that the cells exiting the proliferation compartment due to competition (term 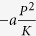 in the equation above) enter the necrotic compartment, we obtain the following differential equations (endowed with initial conditions):


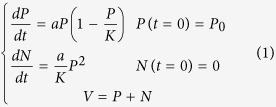


where *P*_0_ is the number of cells injected, converted into a bioluminescence signal using the aforementioned cell-to-light ratio and the rule 10^9^ cells ≅1 cm^3^.

The model was fit to the data using the nonlinear mixed-effects statistical framework for dealing with the non-negligible inter-animal variability (see Methods). It was fully able to fit the bioluminescence data and quantified a plateau reached at 4.35 × 10^9^ ± 2.40 × 10^9^ photons/sec (Fig. 6b). Identifiability of the parameters was excellent, as revealed by low standard errors (Table 1). Based on these parameters retrieved from the bioluminescence data, simulation of the full model kinetics predicted a biphasic pattern for the tumor volume growth curve: first an exponential phase, followed by a linear phase (Fig. 6c). Interestingly, this prediction was both qualitatively and quantitatively verified in the caliper-measured volume experimental data (Fig. 6d). Indeed, the model was able to accurately predict the volume growth kinetics up to a proportionality constant λ that was estimated to 2.25 ± 0.23, after renormalization of the signal into a cm^3^ unit using the cell-to-signal ratio determined above and the approximation 1 cm^3^ ≅ 10^9^ cells (Fig. 6d). We hypothesize that this constant λ is associated to the reported fact that not the entire tumor volume is composed of tumor cells (i.e., alive plus necrotic) as it also comprises a non-negligible part of stroma (estimated here to 56% of the total volume). Taken together, our results show that our simple mathematical model is able to describe the dynamics of both the proliferative component and the entire tumor volume. In doing so, it provides a valid explanation of the exponential-linear pattern of the tumor volume kinetics.

## Discussion

Noninvasive imaging of tumor-bearing rodents has brought a real improvement in experimental therapeutics in oncology, especially with animal models for which tumor and metastasis growth cannot be evaluated with a standard caliper because most lesions are too deep to be accessible by external measurement without animal killing. Optical imaging, and particularly bioluminescence imaging allows to detect small number of living tumor cells^17^,^18^. The sensitivity of the technique can be further improved by developing alternative systems for making tumor cells emitting light, thus rendering them easily detectable even in deeper tissues such as brain^19^. In addition to the sensitivity issue, when precise quantitative (e.g., size of the lesions) and qualitative data (e.g., number of distinct lesions in a same area and exact localization, i.e. liver metastasis or peritoneal carcinosis) are required, standard 2D bioluminescence imaging presents several limitations, mainly related to photon absorption by water and tumor surrounding tissues that will increase signal diffusion before it is acquired by the camera^20^. Indeed, whereas bioluminescent sources on the skin or closely located under the skin of an animal can be precisely localized and quantified, the actual position and precise quantification of deep sources cannot be calculated anymore from surface measurements, due to optical scattering and tissue absorption issues. As a result, 2D imaging can provide only semi-quantitative data, and poor resolution with respect to the exact localization of the tumor cells. Indeed, the most critical information is not related to the emission at the surface, but rather relates to the actual bioluminescence source inside the animal. These important parameters are related to the source brightness, position and geometry^16^. In order to achieve the most accurate measurement, it is therefore necessary to reconstruct precisely the source of the luminescent signal, a requirement that is only achieved by bioluminescence tomography^19^. Bioluminescence imaging system equipped with softwares specifically designed to run 3D algorithms have been made available to achieve such a goal, provided that several wavelengths are used in a row when acquiring the signals. In this study, we evaluated the performances of this new system in terms of reproducibility, reliability and precision. We first demonstrated that the algorithm used by the software for signal reconstruction could be considered as reliable for measuring both primary tumor and secondary lesions. Standard deviation for 10 successive reconstructions was found to be less than 5% and intra-individual variability was not statistically significant. Although not statistically significant either, lung lesions showed a higher variability (i.e. 8%), much probably due to the fact that living imaging of the lung is challenging because of the constant movement of the thorax and the elevated amount of water found in this organ^21^,^22^. However, we found that the 3D reconstruction was highly reproducible and reliable over repeated acquisitions. Finally, detection by DLIT imaging of metastasis regardless of their size was found to be fairly correlated (r^2^ > 0.6) to the actual tumor mass measured after necropsy, even if our data suggest that above 2.5 g, the signal could be less accurate, probably because of blood flow issues and lack of proper homogenous delivery of luciferine in bigger mass with necrotic core and anarchic vasculature. However, animal welfare guidelines usually require that mice are sacrificed when tumors reach 2.5 g in a 30 g mouse and therefore this should not be an issue. When tumor mass is in the 1–2 g range, bioluminescence provides lower signal as compared with mass, much probably because it only addresses living tumor cells whereas weighting tumor encapsulate both living tumor cells with necrotic, calcified and inflammatory tissues. This could explain the differences found later when comparing tumor growth profile monitored using either a caliper or by bioluminescence. DLIT imaging was next compared indeed with reference tumor measurement methods: Caliper and 2D bioluminescence imaging. Caliper was found to be a very convenient tool (cheap, non-invasive, minimal animal manipulation, and easy to use) to evaluate primary tumor growth but it exhibited several limitations: it can be only applied to external tumors, and as for weighting removed tumors it cannot discriminate actual proliferative cells from necrotic, inflammatory or calcified tissues, and does not allow monitoring deep metastasis. Moreover, caliper remains a subjective measurement which differs according the operator and the formula used to calculate the tumor volume^23^. In this study, marked differences were observed in the main tumor growth between external measurement with a calliper and both 2D and 3D bioluminescence imaging. Of note, whereas the use of a calliper suggested a long lasting exponential phase of growth over more than 40 days, bioluminescence monitoring showed conversely that living tumor cells stopped to proliferate around Day-20, thus reaching a plateau much earlier than initially thought. Retrospectively, the longer exponential phase measured with a calliper could be attributed to a constantly increasing mass of necrotic or inflammatory tissues, whereas the actual number of living tumor cells remained almost constant. This observation was further supported by a quantitative mathematical modelling analysis that demonstrated that a simple model with only two parameters, based on two minimal hypotheses (H1: tumor is composed of proliferative and necrotic tissues and H2: growth of the former is governed by a competition phenomenon, i.e. logistic growth), could describe simultaneously the bioluminescence and caliper data sets. Additionally, this novel, biologically-based and identifiable model of tumor growth sheds new light on the previously reported biphasic exponential-linear pattern of tumor growth^24^,^25^. Indeed, our model recapitulates this behaviour (Fig. 6c) but from a mechanistic rather than a phenomenological basis, thus giving a possible explanation. In our theoretical framework, when the proliferative tissue reaches saturation, this generates a constant growth rate of the volume (because 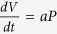), which in turn provokes the transition to the linear phase also observed in our data (Fig. 6d).

In addition, ROI/2D and DLIT/3D bioluminescence analyses did not yield exactly the same profile of primary tumor growth at the end of the experiment. Indeed, after reaching the plateau, tumor seemed to growth again after Day-36 with 2D ROI measurements, whereas no significant increase was evidenced with 3D monitoring after Day-25. We can hypothesize that at the beginning of the experiment only primary tumors were measured, but as the metastasis appeared throughout time, especially in peritoneum area, 2D measurement failed in discriminating properly main tumor from peritoneal carcinosis. Indeed we demonstrated (Fig. 2b,c) that signals from close peritoneal metastasis could merge with main tumor and therefore be mistakenly considered as part of breast primary cancer. Thus, at the end of the experiment combined signals from breast and emerging secondary lesions in the lower peritoneal cavity may have led to overestimating the size of the primary tumor, wrongly suggesting it could grow again after reaching a plateau. At the opposite, DLIT imaging allowed a precise cartography of tumor signal and metastasis as they could be fully distinguished for measurement. To summarize, DLIT imaging enables better distinction between primary tumor and metastasis, with higher accuracy and better reliability than 2D-imaging or caliper measurement and a more accurate estimation of the mass of actually proliferating cells.

## Conclusion

In this study, we showed that monitoring metastasis was feasible without removing the main tumor although it could be a possible massive light emitting source possibly interfering with secondary smaller signals such as disseminated metastasis. Because surgery may affect metastatic spreading on its own^26^, using an imaging technique that allows monitoring precisely tumor invasiveness in real-time with no bias from surgery could be critical, especially in studies investigating on the complex primary tumor-metastasis relationships and experimental therapeutics. Beside, our data suggest that canonical caliper-based measurement of tumors could strongly overestimate exponential phase of primary tumor growth, because it fails to discriminate proliferative from non-proliferative cells. Thus, bioluminescence, especially DLIT imaging, is a state-of-the-art technology in experimental therapeutics in oncology when monitoring both main tumor growth plus multi-metastatic sites is required. In addition, the dedicated mathematical modeling support we present could be much useful to better interpret the data and to avoid misinterpretation of tumor growth kinetics, i.e. by confounding proliferative tumor cells and necrotic or inflammatory tissues.

## Methods

### Animals

The project was submitted to both the French Ministère de l’Education Nationale, de l’Enseignement Supérieur et de la Recherche (MENESR) and the Comité d’Ethique (#CE14) of Aix- Marseille University and started after approval. Pathogen-free 6-week-old female Nod Scid Gamma mice (Charles River laboratories, Lyon, France) were acclimatized in a sterile environment for 2 weeks before starting the study. Mice were maintained in sterilized filter-stopped cages throughout the experiments. All animals were handled and cared in accordance with European rules, based on the UKCCCR guidelines for the welfare of animals in experimental neoplasia^27^. They were daily monitored for signs of distress, decreased physical activity or any behavioral change and weighted thrice a week. Water was supplemented with paracetamol (acetaminophen, equivalent 80 mg/kg/day) to prevent metastasis-related pain^28^.

### Cell culture

The human breast cancer, LUC+ stably transfected, MDA-MB-231-luc-D3H2LN, was obtained from Perlin Elmer (Hopkinton, MA, USA). These adherent cells were grown in RPMI-1640 medium (Invitrogen, CergyPontoise, France) supplemented with 2mM L-glutamine (Invitrogen, France), 5 IU/mL penicillin streptomycin (Eurobio, France), 0,1 IU/mL fungizon (Eurobio, France) and 10% of fetal calf serum (Eurobio, France) at 37 °C in a humidified atmosphere with 5% CO_2_.

### Cells-to-light ratio determination

In order to establish the relationship between a single cell and the number of photons emitted per second (photons/sec), MDA-MB-231-luc-D3H2LN cells were seeded at various densities (i.e., 100 to 10^6 ^cells/well) in a 24-well plate. Fifty μl of a 12 mg/ml solution of Luciferine was then added to the medium and imaging was immediately performed using the IVIS spectrum imager (Perkin Elmer, France) equipped with the Living image 4.3.2. software.

### Preparation of cell suspensions and orthotopic cell implantation

Before implantation, MDA-MB-231 were trypsinized, counted and centrifuged (5 min, 2500 rpm −4 °C) and washed twice in sterile Phosphate Buffered Saline (PBS). Cells were resuspended in RPMI-1640 with 60% of Matrigel^®^ (BD sciences, France) and placed into ice until injection. Mice were placed under gas anesthesia (Isofluran 2%, Abbott France). A volume of 50 μL containing 1.5 × 10^5^ cells were injected in the mammary inguinal left fat pad through the nipple. All applicable institutional and/or national guidelines for the care and use of animals were followed.

### Bioluminescence imaging

Imaging and recording were started at day-6 post-graft until day-43 in order to record the signal emitted from the engrafted tumor and metastasis. Bioluminescence measurements were performed twice a week. Intraperitoneal injection of D-Luciferin firefly (Perkin Elmer, France) at a dose of 150 mg/kg body weight was performed on animals. Imaging and data processing were performed using the IVIS Spectrum imager equipped with the Living Image^®^ 4.3.2 software (Perkin Elmer, France). Acquisitions started 12 minutes after injection, in animal under gas anesthesia (Isoflurane 2%). For 3-D bioluminescence, images were measured between 560 and 660 nm emission wavelengths with a 20 nm-step. Based upon the tomography 3D print of the animal, it was possible, using a built-in anatomic atlas, to simulate the place of main organs. For comparison purpose, mice were monitored using both 3D and 2D imaging procedures in a row. Similarly, external volume of the primary tumor was evaluated using a Vernier caliper the same day than the imaging procedure using the standard V = (lxw^2^)/2 Carlsson formula^23^.

### Necropsy procedures

Mice were sacrificed according to humane killing methods (i.e. cervical dislocation) and body weight was determined at day 43 post-engraftment. The whole body was examined in order to search metastasis in different organs and a new bioluminescent acquisition was immediately performed. Then, both tumor and metastasis were removed, isolated from surrounding tissues under binoculars and weighed.

### Modeling and Statistical Analysis

Inter-group differences were tested using One-Way Anova with Tukey’s HSD Post Hoc multiple comparison testing or Anova on Ranks with Dunn’s method, according to data distribution using Sigma Stat 4.0. software (Systat Software, Germany). An original mathematical model was developed to describe the data. For practical reasons, the model was kept as simple as possible with a limited number of parameters to allow next reliable estimations. For the model fits, parameters estimation was conducted using statistical nonlinear mixed-effects modeling^29^. This approach is particularly well suited for modeling of longitudinal data in a population of subjects. Briefly, instead of assuming independent values of the parameter sets for each animal of the population, it is based on the hypothesis that the individual parameter values are issued from the same population distribution, modeled with a parametric distribution (assumed here to be lognormal). Denoting *θ* = (*a*, *K*) the parameters vector, the statistical model for comparison of the mathematical model to the data thus writes


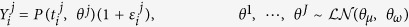


where 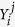 is the data point of individual *j* at time 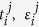 are the residual errors assumed to be independent and identically distributed (proportional error model). The vector *θ*_*μ*_ is the vector of fixed effects and the matrix *θ*_*ω*_ is the covariance of inter-animal random effects. Inference of these population parameters was performed by maximization of the likelihood using the SAEM algorithm^30^ implemented in the Monolix software (Monolix 2016R1, Lixoft). The resulting values are reported in the Table 1.

## Additional Information

**How to cite this article**: Mollard, S. *et al. In Vivo* Bioluminescence Tomography for Monitoring Breast Tumor Growth and Metastatic Spreading: Comparative Study and Mathematical Modeling. *Sci. Rep.* **6**, 36173; doi: 10.1038/srep36173 (2016).

**Publisher’s note:** Springer Nature remains neutral with regard to jurisdictional claims in published maps and institutional affiliations.

## Figures and Tables

**Table 1:** Values of the parameter estimates resulting from fitting the model to the data.

Parameter	Unit	Estimate (CV)	RSE (%)
a	day^−1^	0.399 (31.1)	7.02
K	photons/second	4.35 × 10^9^ (55.3)	12.8
λ	—	2.25 (10)	4.65

RSE = Relative Standard Error. CV = Coefficient of inter-animal Variability expressed in percents.
